# Depression and stress levels in patients with different psychiatric disorders during concurrent early-phase COVID-19 pandemic and earthquake in Croatia

**DOI:** 10.1186/s12888-023-05302-w

**Published:** 2023-11-01

**Authors:** Marina Šagud, Maja Bajs Janović, Zrinka Vuksan Ćusa, Nenad Jakšić, Lucija Bagarić Krakan, Dražen Begić, Jasmina Grubišin, Špiro Janović, Saša Jevtović, Biljana Kosanović Rajačić, Gloria Mamić, Suzan Kudlek Mikulić, Darko Marčinko, Alma Mihaljević Peleš, Maja Šeparović Lisak, Zoran Štimac, Maja Živković, Bjanka Vuksan Ćusa, Wei Wang

**Affiliations:** 1 School of Medicine, University of Zagreb, Zagreb, Croatia; 2 Department of Psychiatry and Psychological Medicine, University Hospital Centre Zagreb, Zagreb, Croatia; 3 University Centre Varaždin, University North, Varaždin, Croatia; 4 Health Centre “Rudeš”, Zagreb, Croatia; 5 Department of Psychology, Norwegian University of Science and Technology, Trondheim, Norway

**Keywords:** Schizophrenia spectrum disorders, Depression, Anxiety, Stress, Earthquake, Covid-19 pandemic, Lockdown

## Abstract

**Background:**

While Croatia shared COVID-19 pandemic with other countries, its capital area was also hit by a 5.6 magnitude earthquake. The simultaneous impact of these two disasters on psychiatric patients is largely unknown, and we addressed those knowledge gaps.

**Methods:**

The cross-sectional study was conducted during the pandemic’s first peak, in the aftermath of earthquake, by telephonic survey. Measurements included the Patient Health Questionnaire-9, the Perceived Stress Scale and the semi-structured interview to evaluate the impact of pandemic stress and earthquake. Overall 396 patients with depression and/or anxiety disorders (DAD), 229 participants with schizophrenia spectrum disorders (SSD) and 205 healthy controls were enrolled.

**Results:**

Both patient groups had higher depression and stress levels than controls, independent of sex, age and the presence of somatic comorbidity. After controlling for the same covariates, patient groups had higher COVID-19- and earthquake-related fears than controls. In patients with DAD, both fears were greater than among SSD patients. When comparing the two fears, the fear from earthquake was higher in DAD and control groups, whereas in SSD patients there was no such difference.

**Conclusions:**

Patients with DAD were the most vulnerable group during disasters, while earthquake seems to be associated with more fear than the pandemics, at least in DAD patients and healthy individuals. Future longitudinal studies should determine if early psychological support might alleviate stress levels after disasters and prevent further worsening of mental health, particularly among DAD patients.

## Background

Traumatic events such as pandemics and earthquakes are often unpredictable. The response to highly stressful events is highly individual, depending on the interaction between psychobiological vulnerability, duration and intensity of stressors and the environmental support. In general, disasters disproportionately affect patients with serious mental illness [1], due to impaired tolerance for adversity and often poor psychosocial background. Consequently, when the entire population is exposed to the same natural disaster, psychiatric patients might have more detrimental outcomes than general population.

### The impact of pandemic on psychiatric patients

The coronavirus disease 2019 (COVID-19) pandemic represents a worldwide challenge. Abundant literature explored the effects of COVID-19 pandemics on mental health in diverse populations, such as general population [2–4] and healthcare providers [5, 6]. A global depressive symptom prevalence of 21.4% during the COVID-19 pandemic was higher than an aggregate point prevalence of 12.9% before the pandemic [7]. Unfortunately, mental health consequences of COVID-19 were found to be comparable to major disasters and armed conflicts [8]. This pandemic had particular impact on patients with severe mental illness. They had higher COVID-19-related mortality than non-psychiatric population [9]. Moreover, many activities have stopped and individuals have been forced to stay at home for prolonged periods, causing disruptions in personal connections and usual behavioral patterns, which may particularly affect this highly vulnerable population.

### The impact of earthquake on psychiatric patients

Earthquakes have also impact on general population mental health. For example, a meta-analysis of studies conducted after earthquakes of magnitude on Richter scale ranging from 4.3 to 9.0, reported the incidence of PTSD in 23% survivors [10]. Another meta-analysis has found severe PTSD symptoms in 42.3%, and depression in 32.1% of individuals following the Haiti 2010 earthquake [11], as well as depression in 22.2% of Chinese people who experienced a devastating earthquake in 2008 [12]. However, much less is known about the impact of earthquake on individuals who already had psychiatric disorders. Several studies reported increased use of psychiatric services after an earthquake at least in some clinical populations [13–15]. Despite different settings, regarding earthquake magnitudes, exposure frequency, socioeconomic conditions and the availability of mental health resources, these findings imply that earthquakes had effects on the mental health service utilization.

While the impact of COVID-19 on psychiatric patients has been extensively studied, only few data exist regarding the earthquake effects. The majority of studies addressed the prevalence of earthquake-related psychiatric disorders in the exposed population [16]. However, only few studies have addressed the effects of earthquake on patients with pre-existing psychiatric disorders. Three studies have assessed the severity of psychopathology before and after the earthquake. The first study demonstrated worsening of positive and cognitive symptoms in first-episode, but not in chronic schizophrenia patients, after L’Aquila 6.3 magnitude earthquake [17]. Another study after the same earthquake has shown a better self-reported outcome in patients with schizophrenia and mood disorders, but negative consequences in those with anxiety disorders [18]. The third study detected coping difficulties and elevated stress reactivity following the Northridge Earthquake of 6.9 magnitude, in patients with schizophrenia than in healthy controls and the intermediate difficulties among bipolar patients [19]. Studies were limited by small sample sizes, but suggested that the impact of earthquake on psychiatric population was not uniform [17–19].

### The impact of both disasters on psychiatric patients

At the end of the fourth week of the epidemic in Croatia, when the experts thought that nothing worse could happen, on 22 March 2020, the citizens of Zagreb were awakened by an earthquake of 5.5 magnitude on the Richter scale, followed the 57 aftershocks in the next 24 h, and the extensive property damage [20], with 27 people being severely injured, of which one died [21]. Mutual impacts of the earthquakes and the pandemic represent a dual psychological challenge [22]. Several studies in Croatia addressed the impact of both disasters, by comparing their effects between exposed and non-exposed groups. While three studies reported increased psychological damage in non-clinical populations exposed to both disasters compared to people who experienced only pandemic [23–25], and one did not [26], there is no data on the impact of those two disasters on psychiatric patients.

Although the COVID-19 era has been associated with 7 major earthquakes so far [22], to best of our knowledge, there are no reports on the effects of concurrent earthquake and pandemic on psychiatric patients. Moreover, diverse diagnostic groups might react differently, depending on their existing pathology and coping mechanisms. The present study aimed to compare the impact of earthquake and COVID-19 pandemic on perceived stress, symptoms of depression, COVID-19-related fear and earthquake-related fear, between (1) patients with schizophrenia-spectrum disorders, (2) affective and/or anxiety disorders, and (3) non-psychiatric population.

## Methods

### Subjects

This cross-sectional study included control and patient groups. Inclusion criteria for both patient and control groups were: (1) aged 18 years or older, (2) provided consent to participate, (3) not employed in health-care (due to increased burden to health-care workers in Covid-19 era). Exclusion criteria for both groups were: (1) having current or recent severe somatic or neurological condition which represents significant burden, (2) ongoing other severe stressors other than pandemics and earthquake, such as being in the bereavement, severe medical condition in family member or ongoing the divorce.

This study included patients who received care at Department of Psychiatry and Psychological Medicine, University Hospital Center Zagreb, and who were scheduled for outpatient visit. While all outpatient visits were canceled during the quarantine, patients were given a phone call by their psychiatrist to call off the session, and to check how the patient was doing in times of both pandemic and earthquakes. Inclusion criteria for patient groups were: 1) being diagnosed and currently treated as outpatients for depressive and/or anxiety disorders (DAD) or schizophrenia-spectrum disorders (SSD) in the Department of Psychiatry and Psychological Medicine. Exclusion criteria for patient groups were: 1) Being hospitalized at a psychiatric department after the onset of pandemics, which includes the timing of the earthquake, and 4) not having sufficient capacity to conduct the interview, i.e., being diagnosed with mild cognitive disorder, dementia or intellectual difficulties, or currently with severe psychiatric symptoms. Patient diagnoses were previously verified by the attending psychiatrist according to the International Classification of Diseases (ICD) 10th revision (ICD-10) [27], which was evident from electronic medical records. If patients had more than one psychiatric diagnosis, the first one was mentioned. Eligible patients were asked for consent and telephone survey was conducted. All participants were interviewed during a single phone call. Each patient was interviewed by her or his psychiatrist, who started treating the patient before the pandemics and was well-known to the patient. None of the psychiatric patients refused to take part in this study.

Control group was recruited in the Health Centre „Rudeš“ in Zagreb. Specific inclusion criterion for the control group was: 1) not having past or present psychiatric disorder. Recruited were the patients who called general practitioner office for different reasons, such as asking advice. Eligible patients were offered to participate in the study. Only 3 non-psychiatric patients refused to take party in this study due to personal reasons.

### Procedures

Participants completed the Patient Health Questionnaire-9 (PHQ-9), which is used to screen for symptoms of depression in the past 14 days. A PHQ-9 total score of ≥ 5 was considered as “having depression”, and ≥ 10 was considered as “having moderate to severe depression” [28]. The Patient Health Questionnaire (PHQ) was identified as the most commonly reported standardized depression metric in the extant COVID-19 literature [7]. The internal consistency in this study was adequate, as seen in the Cronbach’s alpha coefficient of 0.83.

Perceived stress was assessed using the „Perceived Stress Scale“ (PSS-10). Moderate or high perceived stress was assessed using cutoff scores of ≥ 14 for the PSS-10 [29, 30]. It is composed of 10 questions, with each being rated on a 5-point Likert scale, from 0 (never) to 4 (very often) during the last month. It includes 6 positively (items 1, 2, 3, 6, 9 and 10: Positive factor) and 4 negatively (items 4, 5, 7 and 8: Negative factor) worded items. Negative worded items were re-coded during analysis by the following principle: 0 = 4, 1 = 3, 2 = 2, 3 = 1, 4 = 0 [29]. Total scores range from 0 to 40, with higher scores indicating greater levels of self-rated stress. In our sample this scale had adequate internal consistency as reflected in the Cronbach’s alpha coefficient of 0.89.

A semi-structured questionnaire was designed for this study with the following questions to evaluate the impact of pandemic stress and earthquake:

To evaluate the impact of pandemic stress in clinically stable patients, we used a semi-structured interview in which we asked: (a) if the fear of being infected was not at all/a little bit or moderately/quite a bit or distressing; (b) if changes in lifestyle, such social isolation, related to quarantine were not at all/a little bit or moderately/quite a bit distressing, (c) if the fear of earthquake was not at all/a little bit or moderately/quite a bit or distressing; (d) if changes in lifestyle, related to earthquake (such as sleeping with earthquake emergency bag) were not at all/a little bit or moderately/quite a bit distressing. Answers were scored using the 1 to 5 Likert scale, with higher scores denoting higher levels of distress (1, not at all; 2, a little bit; 3, moderately; 4, quite a bit; 5, distressing).

### Statistical analysis

Statistical analyses were carried out in the computer software SPSS, version 20. Additionally, we used the G.Power (version 3.1) software to determine the post-hoc statistical power of this study; comparison via ANCOVA of the three samples (N = 830, f = 0.25, 3 groups, 4 covariates) yielded a very high statistical power of 98%. Internal reliabilities of the PHQ-9 and PSS-10 scales were expressed via the Cronbach’s alpha coefficients. Descriptive statistics included arithmetic means (M), standard deviations (SD), ranges and percentages. Comparison between the three groups in nominal variables (sex, marital status, employment status) was conducted using the chi-square test. The same comparison in continuous variables was conducted using the one-way ANOVA (age) and one-way ANCOVA (PHQ-9 depression, PSS-10 stress, COVID-19 distress and lifestyle restrictions, earthquake distress and lifestyle restrictions), while controlling for the influence of sex, age, presence of somatic comorbidity and earthquake-related household damage in the case of ANCOVA. Post-hoc Scheffe test was used for examining differences between each pair of groups. Finally, to determine whether potential differences exist between pandemic-related and earthquake-related fear within the three groups, t-tests for dependent-samples were performed. Statistical significance was set at p = .05.

## Results

### Sociodemographic data

Overall, 846 participants were included. Among them, 16 provided insufficient information, and 830 respondents have completed all data, inclusive of 396 patients with DAD, 229 patients with SSD and 205 control respondents. The mean age of DAD patients was 52.61 ± 13.19 years (age range: 19–85), mean SSD patient age was 47.51 ± 12.80 years (age range: 22–76), whereas mean age of the control group was 46.57 ± 15.23 years (age range: 19–81). One-way ANOVA detected significant differences in age (F = 17.40, p < .001). Post hoc Scheffe test has shown that DAD patients were significantly older than those in SSD and control group, while there were no differences between participants in SSD and control group.

Other sociodemographic data are presented in Table 1.

**Table 1:** Sociodemographic characteristics of the study participants

Variable		SSD N (%)	DAD N (%)	Control group N (%)	*χ* ^2^	p
Sex	Male	77 (33.6%)	179 (45.2%)	98 (47.8%)	10.908	0.004
	Female	152 (66.4%)	217 (54.8%)	107 (52.2%)		
Marital status	Single	110 (48.1%)	57 (14.4%)	38 (18.5%)	116.513	0.000
	Civil partnership	14 (6.1%)	33 (8.3%)	24 (11.7%)		
	Married	77 (33.6%)	250 (63.1%)	122 (59.5%)		
	Widowed	9 (3.9%)	20 (5.1%)	18 (8.8%)		
	Divorced	19 (8.3%)	36 (9.1%)	3 (1.5%)		
Employment status	Employed, currently working	41 (17.9%)	106 (26.8%)	111 (54.1%)	115.849	0.000
	Employed, on sickness leave	28 (12.2%)	63 (15.9%)	40 (19.5%)		
	Unemployed	46 (20.1%)	43 (10.9%)	14 (6.8%)		
	Retired	106 (46.3%)	168 (42.4%)	26 (12.7%)		
	Student	8 (3.5%)	16 (4%)	14 (6.8%)		

DAD – depressive and/or anxiety disorders; SSD – schizophrenia-spectrum disorders; χ^2^ – chi-square test; p – statistical significance

The differences between groups were tested using three χ^2^ – chi-square tests. While DAD and control groups had similar proportion of males and females, females were overrepresented in SSD group. Respondents in DAD and control group were more frequently married than those in SSD group, while SSD group contained more singles. Participants in the control group were more frequently employed compared to the rest of the groups, while SSD patients were the most frequently unemployed. Individuals from both patient groups were more commonly retired than participants from the control group.

Table 2 provides the list of patient diagnoses, according to the ICD-10 classification.

**Table 2:** The psychiatric diagnoses of the study participants

SSD		DAD	
Diagnosis	Frequency (%)	Diagnosis	Frequency (%)
Schizophrenia (F20)	108 (47.2%)	Bipolar disorder (F31)	33 (8.3%)
Schizotypal disorder (F21)	2 (0.9%)	Depressive episode (F32)	45 (11.4%)
Delusional disorders (F22)	21 (9.2%)	Major depressive disorder, recurrent (F33)	111 (28%)
Brief psychotic disorder (F23)	20 (8.7%)	Generalized anxiety disorder (F41.1)	4 (1%)
Schizoaffective disorders (F25)	32 (14%)	Mixed anxiety and depressive disorder (F41.2)	66 (16.7%)
Unspecified psychosis not due to a substance or known physiological condition (F29)	46 (20%)	Obsessive-compulsive disorder (F42)	3 (0.8%)
		Post-traumatic stress disorder (PTSD) (F43.1)	87 (22%)
		Adjustment disorders F43.2	36 (9.1%)
		Dissociative and conversion disorders (F44)	6 (1.5%)
		Somatoform disorders (F45)	5 (1.2%)

DAD – depressive and/or anxiety disorders; SSD – schizophrenia-spectrum disorders

### Clinical differences across the groups

Table 3 represents the differences between the severity of depressive and stress symptoms, and the extent of COVID-19- or earthquake-related disturbances and lifestyle restrictions.

**Table 3:** The comparison between groups regarding symptoms of depression and stress, and the impact of COVID-19 and earthquake on feelings of discomfort and lifestyle restrictions

	SSD	DAD	Control group		
	M ± SD	M ± SD	M ± SD	F	p
Severity of depression (PHQ-9)	7.97 ± 5.94	8.96 ± 6.57	4.05 ± 2.98	19.797	0.000
Stress level (PSS-10)	20.47 ± 6.78	21.57 ± 6.42	14.11 ± 6.16	32.897	0.000
COVID-19**-**related distress	2.31 ± 0.94	2.50 ± 0.90	2.02 ± 0.80	13.120	0.000
COVID-19-related lifestyle restriction	2.43 ± 0.93	2.63 ± 0.89	2.76 ± 0.93	6.888	0.000
Earthquake-related distress	2.44 ± 1.06	2.64 ± 1.01	2.18 ± 0.91	13.490	0.000
Earthquake-related lifestyle restriction	2.03 ± 1.00	2.36 ± 1.10	1.56 ± 0.95	25.172	0.000

DAD – depressive and/or anxiety disorders; SSD – schizophrenia-spectrum disorders; M – arithmetic mean; SD – standard deviation; F – Fisher’s ratio; p – statistical significance

One-way analysis of covariance (ANCOVA) was used to compare aforementioned variables across the three groups, while controlling for other variables (covariates) which may influence the results. There was a significant difference between groups in the severity of depression, which was independent of sex, age, the presence of somatic comorbidity and earthquake-related household damage. Post-hoc test revealed higher levels of depression in both patient groups compared to control group (p < .01), while DAD and SSD patients had similar severities of depression (p = .180).

Likewise, significant difference in levels of stress was detected between groups, which was also independent of sex, age, the presence of somatic comorbidity and earthquake-related damage in participant’s homes. Post-hoc test demonstrated greater levels of stress in patient groups compared to control group (p < .01), whereas DAD and SSD patients were similar in this regard (p = .058).

There was a significant difference in the COVID-19-related distress between groups, independent of sex, age, the presence of somatic comorbidity. Post hoc test identified higher COVID-19 stress levels in both patient groups than in controls (p < .01), whereby DAD patients had higher pandemic stress severity than SSD patients (p = .024).

Significant difference in the COVID-19-related lifestyle restrictions between groups, independent of sex, age, the presence of somatic comorbidity, was also found. The comparison of three groups with the post-hoc test reported higher lifestyle restrictions in controls than in SSD (p < .01) and DAD groups (p = .036), while DAD patients displayed greater lifestyle changes than SSD patients (p = .01).

Significant difference in the earthquake-related stress between groups was observed, independent of sex, age, the presence of somatic comorbidity and earthquake-related damage of their homes. Post-hoc test has found higher earthquake-related stress levels in patient groups than in controls (p < .01). Moreover, two patient’s groups also differed, given that DAD patients had higher earthquake-related stress levels than SSD patients (p < .01).

Finally, significant difference in the earthquake-related lifestyle changes between groups was observed, independent of sex, age, the presence of somatic comorbidity and earthquake-related home damage. Post-hoc test has shown greater earthquake-related lifestyle changes in patient groups than in controls (p < .01). In addition, DAD patients had higher earthquake-related lifestyle changes than SSD patients (p < .01).

To determine whether potential differences exist between pandemic-related and earthquake-related fear within groups, t-test for dependent-samples was performed, as presented in Table 4.

**Table 4:** The comparison of COVID-19 related and earthquake-related fear between patients with DAD, patients with SSD and controls

	COVID-19-related fear	Earthquake-related fear	p
Patients with SSD	2.31	2.44	0.054
Patients with DAD	2.50	2.64	0.003
Control group	2.02	2.18	0.028

DAD – depressive and/or anxiety disorders; SSD – schizophrenia-spectrum disorders; p- statistical significance level

Patients with DAD reported significantly higher, albeit small, earthquake-related fear than COVID-19-related fear (p = .003). Similarly, participants from control group reported small yet significantly higher earthquake-related fear than COVID-19-related fear (p = .028). Conversely, SSD patients exhibited no significant difference between earthquake- and COVID-19-related fears (p = .054).

## Discussion

The main findings of the present study are that (1) Patient groups had higher depression and stress levels than controls, but depression and stress levels were similar in patients with DAD and SSD, (2) Patient groups also had higher COVID-19- and earthquake-related fears than controls, while both fears were greater in DAD than SSD patients, and (3) the fear from earthquake was slightly, but significantly higher than the fear from COVID-19 infection in DAD and control groups, whereas in SSD patients there was no such difference.

### COVID-19-related fear

This study was conducted in the early pandemic phase, when the number of infected individuals was very low, so the results of higher COVID-related fear cannot be explained by biological effects of infection, or acute stress or bereavement due to consequences of infection. It could be rather attributed to the psychological effects of „lock-down “, such as restriction in social contacts and care [31]. This may constrain usual coping mechanisms, such as seeking comfort from the closest ones and/or escape to available “safe spots”. In agreement, in the early pandemic phase, more loneliness, low coping, insufficient treatment and pandemic worry predicted poor mental health [32]. Physical distance may also increase feelings of loneliness and sudden isolation in this fragile population [1]. Patients who live alone might be particularly affected by isolation.

Many studies have assessed the COVID-19 impact on psychiatric populations. The majority of them have demonstrated more pronounced psychological symptoms in psychiatric patients, and those with affective disorders were the most frequently affected, although some reported a reduction of psychiatric symptomatology [33]. For example, in accordance with our results, patients with affective disorders claimed to be more negatively affected by COVID outbreak than participants with psychotic disorders [34]. Likewise, among participants with a history of mental illness, those with anxiety, depression, post-traumatic stress disorder or eating disorder were more likely to report the worsening of mental health during the pandemic than individuals with other diagnoses [35].

In agreement, patients suffering from different psychiatric disorders had more symptoms related to anxiety and depression when compared to control group during the lockdown in Spain [36]. Moreover, participants with psychotic disorders had poorer wellbeing and mental health than bipolar patients [32]. Moreover, COVID-19 outbreak was associated with a higher anxiety response in people with schizophrenia and bipolar disorder than in healthy controls [37]. However, those studies [32, 37], did not include patients with major depression, whereas in our DAD sample, only 8% of patients had bipolar disorder.

Our findings are inconsistent with the Korean study, which reported that patients with schizophrenia had lower levels of fear and COVID-19-associated stress than the general population, despite displaying higher levels of loneliness and depression [38]. Our patients with schizophrenia had higher COVID-19 related fear than controls, despite the fact that they had the lowest life-style changes compared to rest of the groups. In contrast to rest of the groups, the majority of our patients with schizophrenia were unmarried, and only 18% were currently working. Another study found that patients with schizophrenia had similar levels of anxiety and depression as healthy controls early in pandemics, but the same patients reported higher levels of depression and anxiety 6 months later [39]. Those findings suggest that psychological symptoms related to COVID-19 fluctuate over time. Similarly, patients with major depression, bipolar disorder, schizophrenia or schizoaffective disorder did not differ in subjective COVID-19-related fear [40].

Potential sources of discrepancies across studies may arise from the specific circumstances in our study, differences in the pharmacological treatment, and the treatment compliance. The largest difference is that, unlike in all aforementioned studies, all our patients were recently exposed to the earthquake. The minority of our patients was shifted to tele-health at that time, whereas traffic restrictions due to earthquake may have further lowered the availability of usual medical attention. For example, while patients with the history of depression remained stable during early pandemic, those unable to access psychiatric care experienced increase in stress and anxiety [41]. Similar findings in pandemic were observed in schizophrenia, given that those with decreased family support had increased risk for worsening of symptoms [42]. Although the present study did not measure specific disease symptoms, patients had higher levels of stress and anxiety, than controls. However, those differences were not marked. Of note, average depression level in patients with DAD and SSD was mild, while participants from the control group had minimal depression. All our study groups had moderate stress levels, but the differences between clinical and control groups were still significant.

Although the effects of antipsychotics on COVID-19 related fear are completely unexplored, there is evidence on the efficacy of some atypical antipsychotics in the treatment of nonpsychotic anxiety [43]. There is no data on the influence of antidepressants or benzodiazepines on COVID-related anxiety and depression. All our patients were medicated at the time of the interview, and the potential drug effects such as sedation may have alleviated stress response. On the other hand, pandemic circumstances may be associated with decreased compliance and consequent increase in psychopathology, at least in patients with schizophrenia [42], although these findings are equivocal [44].

### Earthquake-related fear

Although both being disasters, pandemics and earthquakes may exert somehow different effects on mental health. For example, social restrictions during COVID-19 pandemic were associated with increased symptoms of depression, stress and loneliness, although not anxiety [45]. However, earthquakes are well-known to cause anxiety [11], including the Croatian earthquake [46, 47], along with the depression and distress [11, 47]. Earthquakes represent the imminent threat to public safety which is uncontrollable and impossible to escape. Unlike pandemics, in which certain protective measures may markedly lower the risk of infection, earthquakes are completely unpredictable, leading to potentially higher levels of uncertainty than pandemic. The tolerance to the increasing levels of uncertainty is diminished in psychiatric patients. Especially close links have been depicted between intolerance to uncertainty and depression, accompanied by rumination and anxiety [48].

### The impact of pandemic and earthquake

Our findings suggest that psychiatric patients have been disproportionately affected by the simultaneous occurrence of earthquake and COVID-19 pandemic. According to other studies carried out in Croatia, earthquake represented additional load to individuals already affected by pandemics, in an online survey in participants from various social network channels, those exposed to earthquake had slightly, but significantly higher levels of stress, anxiety and depression [24]. Another study, conducted on members of the Facebook group dedicated to pandemics in Croatia, reported that the subjects who also experienced the earthquakes showed a higher degree of anxiety and stress, although no depression, than other respondents [25]. Likewise, among patients with atopic dermatitis, those exposed to both disasters, had greater levels of perceived stress compared to participants who experienced only pandemics [23]. Those findings collectively suggest that earthquake represented additional burden to the non-clinical population in the affected region, as reflected in elevated levels of stress-related symptoms. In disagreement, among family medicine health-care workers in Croatia, working in the area hit by an earthquake did not affect their psychological symptoms [26]. However, health-care workers already had high psychological burden, given their prevalence rates of 30.9%, 33.1%, 30.7%, 33.0% of stress, anxiety, depression and clinically relevant score for PTSD, respectively [26], which were even higher than meta-analytic prevalence estimates for health care workers during the COVID-19 pandemic [5]. Therefore, the ceiling effect may have been obtained due to already maximal engagement to provide adequate patient care and protection during the lock-down. In contrast to aforementioned studies, the present study (1) included only participants from the broader Zagreb region, which was affected by earthquake, and (2) was the first one to also include psychiatric patients. It emphasizes that earthquake had larger psychological impact than pandemic regardless of having psychiatric disorder. However, those effects were stronger in psychiatric patients, with patients with affective / anxiety disorders expressing the highest fear from both threats. Our findings are consistent with the past research following disasters, ranging from floods to nuclear disasters to COVID-19, which reported that people with psychotic disorders had poor coping and adverse psychiatric outcomes [49].

The overrepresentation of females in our SSD sample, not corresponding to the epidemiology of schizophrenia, deserves comment. First, slightly less than half of SSD patients were diagnosed with schizophrenia, and the preponderance of females may arise from other SSD categories. For example, schizoaffective disorder is almost twice as prevalent in women compared to men [50]. There is also evidence that delusional disorders are more frequent in females [51]. Second, only patients considered eligible were asked to participate. While among patients with schizophrenia, females have more favorable outcome [52, 53], investigators may have selectively considered females with schizophrenia more appropriate for the telephone interview. Third, robust sex differences were reported in the schizophrenia risk distribution across the age span [54]. While the incidence of schizophrenia is higher in males, they are typically diagnosed earlier, with peak incidence around 22 years, in contrast to females, in whom the incidence declines very slowly between age 18 and 65 [55], and, unlike males, females have the second peak incidence at age 30–39 years [54]. In agreement, women with SSD were older at the first the visit to a mental health service (43.7 vs. 47.8 years) [50]. Given that the mean age of our SSD patients was 47.51 ± 12.80 years (age range: 22–76), more females in this age would have been expected to have schizophrenia, than if younger population was included.

### Limitations of the study

The study was cross-sectional, and there was no data on the depressive and stress symptoms before the occurrence of pandemic and the earthquake. Therefore, it is unknown whether higher levels of stress and anxiety detected in our patients were due to existing psychopathology, or rather due to effects of two simultaneous disasters. Longitudinal studies could provide a more thorough assessment of symptoms and fears related to these two stressful events, as well as their changes over time. However, this study could not have been performed in a longitudinal fashion using the same methodology. Namely, interview was conducted by phone, because outpatient visits were canceled due to quarantine, introduced just several days prior to earthquake (which occurred on March 22nd, 2020). The “lock-down” lasted only one month. Thereafter, the restrictions were relaxed, and in May, 2020, Croatia was among the most liberal EU countries regarding anti-COVID measures. While the patients were interviewed during the first 4 weeks following earthquake, after that period the vast majority of them started visiting their psychiatrists again. However, patients with DAD expressed the highest levels of stress due to new-onset disasters. More specifically, they had higher levels of both COVID-19- and earthquake-related stress, than healthy controls or patients with SSD. This finding suggests that DAD patients are a particularly vulnerable population during times of disaster. Another limitation is that patients were suffering from different depressive and anxiety disorders, which were not separately analyzed. In addition, levels of support, resilience and loneliness were not assessed. Mental stability during COVID-19 pandemic was associated with resilience, better tolerance of uncertainty, less social isolation, while mentally volatile individuals had enhanced pandemic-related worry compared to those who were mentally stable [56]. The generalization of our findings should be limited to lockdown (early) pandemic phase, given than mild improvement in symptoms may occur after re-opening, except for patients with schizophrenia [57], while increase in symptoms of depression was reported in healthy controls between April and November 2020 [39]. Moreover, the results may also not apply to patients who are in unstable condition and/or uncooperative.

### Strengths of the study

This study also has some strengths. It compared the level of fear and depressive symptoms of two unrelated, but simultaneous natural phenomena, global pandemics and earthquake. It included relatively large sample size of psychiatric patients from the same geographical area, treated in the same hospital. In addition, patients were interviewed by their psychiatrists, who have been treating them for many years in general, and with whom they had a good therapeutic relationship. So, their answers may be considered authentic. In case of ambiguous responses, additional clarification was required during the interview. Finally, our obtained findings offer novel insight into different psychological reactions to natural disasters of individuals with specific psychiatric disorders.

## Conclusions

Despite some limitations, our findings expand the current knowledge on the impact of natural disasters on psychiatric patients, and highlight the vulnerability of psychiatric patients, particularly those with DAD disorders. However, among “dual psychological pressures” [22], earthquake appears to have more profound effects. Complete uncontrollability of earthquake may contribute to such fear-provoking effects. Given that disasters represent challenge the continuation of psychiatric treatment, psychiatric patients may not be neglected on such occasions. Our findings emphasize the need to strengthen support and protection to psychiatric patients during disasters, to provide the relied of anxiety, tension and depression. Potential methods include increased monitoring and the promotion of alternative mental-health services, such as tele-health. More research is needed to establish the efficacy of those interventions, in order to prevent the harmful long-term consequences of worsening of mental-health.

## Data Availability

The datasets used and/or analysed during the current study are available from the corresponding author on reasonable request.

## References

[1] Druss BG (2020). Addressing the COVID-19 pandemic in populations with Serious Mental Illness. JAMA Psychiatry.

[2] Thygesen LC, Møller SP, Ersbøll AK (2021). Decreasing mental well-being during the COVID-19 pandemic: a longitudinal study among Danes before and during the pandemic. J Psychiatr Res.

[3] Deng X, He M, Zhang J, Huang J, Luo M, Zhang Z (2022). SARS-CoV-Related Pandemic outbreaks and Mental Disorder Risk. J Nerv Ment Dis.

[4] Taylor SC, Smernoff ZL, Rajan M (2022). Investigating the relationships between resilience, autism-related quantitative traits, and mental health outcomes among adults during the COVID-19 pandemic. J Psychiatr Res.

[5] Li Y, Scherer N, Felix L, Kuper H (2021). Prevalence of depression, anxiety and post-traumatic stress disorder in health care workers during the COVID-19 pandemic: a systematic review and meta-analysis. PLoS ONE.

[6] Ghahramani S, Kasraei H, Hayati R, Tabrizi R, Marzaleh MA. Health care workers’ mental health in the face of COVID-19: a systematic review and meta-analysis. Int J Psychiatry Clin Pract. 2022;1–10. 10.1080/13651501.2022.2101927 10.1080/13651501.2022.2101927 35875844

[7] Lee Y, Lui LMW, Chen-Li D, Liao Y, Mansur RB, Brietzke E (2021). Government response moderates the mental health impact of COVID-19: a systematic review and meta-analysis of depression outcomes across countries. J Affect Disord.

[8] Leung CMC, Ho MK, Bharwani AA, Cogo-Moreira H, Wang Y, Chow MSC (2022). Mental disorders following COVID-19 and other epidemics: a systematic review and meta-analysis. Transl Psychiatry.

[9] Fond G, Nemani K, Etchecopar-Etchart D, Loundou A, Goff DC, Lee SW (2021). Association between Mental Health Disorders and Mortality among patients with COVID-19 in 7 countries: a systematic review and Meta-analysis. JAMA Psychiatry.

[10] Dai W, Chen L, Lai Z, Li Y, Wang J, Liu A (2016). The incidence of post-traumatic stress disorder among survivors after Earthquakes: a systematic review and meta-analysis. BMC Psychiatry.

[11] Cénat JM, McIntee SE, Blais-Rochette C (2020). Symptoms of posttraumatic stress disorder, depression, anxiety and other mental health problems following the 2010 Earthquake in Haiti: a systematic review and meta-analysis. J Affect Disord.

[12] Bi Y, Wang L, Cao C (2021). The factor structure of major depressive symptoms in a sample of Chinese Earthquake survivors. BMC Psychiatry.

[13] Matsumoto J, Kunii Y, Wada A, Mashiko H, Yabe H, Niwa S (2014). Mental disorders that exacerbated due to the Fukushima Disaster, a complex radioactive contamination Disaster. Psychiatry Clin Neurosci.

[14] Sakuma A, Ueda I, Rengi S, Shingai T, Matsuoka H, Matsumoto K (2018). Increase in the number of admissions to psychiatric hospitals immediately after the Great East Japan Earthquake. Asia Pac Psychiatry.

[15] Tseng KC, Hemenway D, Kawachi I, Subramanian SV, Chen WJ (2010). The impact of the Chi-Chi Earthquake on the incidence of hospitalizations for schizophrenia and on concomitant hospital choice. Community Ment Health J.

[16] Tang B, Deng Q, Glik D, Dong J, Zhang L (2017). A Meta-analysis of risk factors for post-traumatic stress disorder (PTSD) in adults and children after Earthquakes. Int J Environ Res Public Health.

[17] Pollice R, Bianchini V, di Mauro S (2012). Cognitive function and clinical symptoms in first-episode psychosis and chronic schizophrenia before and after the 2009 L’Aquila Earthquake. Early Interv Psychiatry.

[18] Stratta P, Rossi A (2010). Subjective adjustment of individuals with psychiatric disorders in the aftermath of the L’Aquila Earthquake. Am J Psychiatry.

[19] Horan WP, Ventura J, Mintz J (2007). Stress and coping responses to a Natural Disaster in people with schizophrenia. Psychiatry Res.

[20] Čivljak R, Markotić A, Capak K (2020). Earthquake in the time of COVID-19: the story from Croatia (CroVID-20). J Glob Health.

[21] Atalić J, Uroš M, Šavor Novak M, Demšić M, Nastev M (2021). The M_w_5.4 Zagreb (Croatia) Earthquake of March 22, 2020: impacts and response. Bull Earthq Eng.

[22] Sayfouri N, Heidari M, Miresmaeeli SS (2022). Mutual impacts of the COVID-19 pandemic and the recent Earthquakes: a scoping review of the lessons learned. Disaster Med Public Health Prep.

[23] Lugović-Mihić L, Meštrović-Štefekov J, Pondeljak N (2021). Psychological stress and atopic dermatitis severity following the COVID-19 pandemic and an Earthquake. Psychiatr Danub.

[24] Margetić B, Peraica T, Stojanović K, Ivanec D (2021). Predictors of emotional distress during the COVID-19 pandemic; a Croatian study. Pers Individ Dif.

[25] Matić I, Takšić I, Božičević M (2021). The sense of coherence and subjective well-being as resources of Resilience in the time of stressful situations: COVID-19 outbreak and Earthquakes. Psychiatr Danub.

[26] Vlah Tomičević S, Lang VB (2021). Psychological outcomes amongst family medicine healthcare professionals during COVID-19 outbreak: a cross-sectional study in Croatia. Eur J Gen Pract.

[27] World Health Organization (1993). The ICD-10 classification of mental and behavioural disorders.

[28] Kroenke K, Spitzer RL, Williams JB, Löwe B (2010). The patient health questionnaire somatic, anxiety, and depressive symptom scales: a systematic review. Gen Hosp Psychiatry.

[29] Cohen S, Kamarck T, Mermelstein R (1983). A Global measure of perceived stress. J Health Soc Behav.

[30] Agyapong VIO, Hrabok M, Vuong W, Shalaby R, Noble JM, Gusnowski A (2020). Changes in stress, anxiety, and Depression levels of subscribers to a daily supportive text message program (Text4Hope) during the COVID-19 Pandemic: cross-sectional survey study. JMIR Ment Health.

[31] Jakovljevic M, Bjedov S, Jaksic N, Jakovljevic I (2020). COVID-19 pandemia and Public and Global Mental Health from the perspective of Global Health Securit. Psychiatr Danub.

[32] Barrett EA, Simonsen C, Aminoff SR, Hegelstad WTV, Lagerberg TV, Melle I (2022). The COVID-19 pandemic impact on wellbeing and mental health in people with psychotic and bipolar disorders. Brain Behav.

[33] Fleischmann E, Dalkner N, Fellendorf FT, Reininghaus EZ (2021). Psychological impact of the COVID-19 pandemic on individuals with serious mental disorders: a systematic review of the literature. World J Psychiatry.

[34] Vissink CE, van Hell HH, Galenkamp N, van Rossum IW (2021). The effects of the COVID-19 outbreak and measures in patients with a pre-existing psychiatric diagnosis: a cross-sectional study. J Affect Disord Rep.

[35] Lewis KJS, Lewis C, Roberts A (2022). The effect of the COVID-19 pandemic on mental health in individuals with pre-existing mental Illness. BJPsych Open.

[36] Solé B, Verdolini N, Amoretti S (2021). Effects of the COVID-19 pandemic and lockdown in Spain: comparison between community controls and patients with a psychiatric disorder. Preliminary results from the BRIS-MHC STUDY. J Affect Disord.

[37] González-Blanco L, Dal Santo F, García-Álvarez L, de la Fuente-Tomás L, Moya Lacasa C, Paniagua G (2020). COVID-19 lockdown in people with severe mental disorders in Spain: do they have a specific psychological reaction compared with other mental disorders and healthy controls?. Schizophr Res.

[38] Lee YR, Chung YC, Kim JJ, Kang SH, Lee BJ, Lee SH (2022). Effects of COVID-19-related stress and fear on depression in schizophrenia patients and the general population. Schizophrenia (Heidelb).

[39] Rosa-Alcázar Á, Parada-Navas JL, García-Hernández MD, Martínez-Murillo S, Olivares-Olivares PJ, Rosa-Alcázar AI (2021). Coping strategies, anxiety and depression in OCD and Schizophrenia: changes during COVID-19. Brain Sci.

[40] Brosch K, Meller T, Pfarr JK (2022). Which traits predict elevated distress during the Covid-19 pandemic? Results from a large, longitudinal cohort study with psychiatric patients and healthy controls. J Affect Disord.

[41] Czysz AH, Nandy K, Hughes JL, Minhajuddin A, Chin Fatt CR, Trivedi MH (2021). Impact of the COVID-19 pandemic on adults with current and prior depression: initial findings from the longitudinal Texas RAD study. J Affect Disord.

[42] Bassiony MM, Sehlo MG, Ibrahim EF, Zayed AE, Atwa SA. Assessment of compliance and relapse in patients with schizophrenia before and after the onset of COVID-19 pandemic. Int J Psychiatry Clin Pract. 2022;1–7. 10.1080/13651501.2022.2124175 10.1080/13651501.2022.2124175 36149774

[43] Pies R (2009). Should psychiatrists use atypical antipsychotics to treat nonpsychotic anxiety?. Psychiatry (Edgmont).

[44] McKee KA, Crocker CE, Tibbo PG (2021). Long-acting injectable antipsychotic (LAI) prescribing trends during COVID-19 restrictions in Canada: a retrospective observational study. BMC Psychiatry.

[45] Knox L, Karantzas GC, Romano D, Feeney JA, Simpson JA (2022). One year on: what we have learned about the psychological effects of COVID-19 social restrictions: a meta-analysis. Curr Opin Psychol.

[46] Bajs Janović M, Janović Å, Šeparović Lisak M, Medved S, Ojdanić O, Veronek J (2021). Phantom Earthquake Syndrome - A Pilot Study after Zagreb and Banovina 2020 Earthquake. Psychiatr Danub.

[47] Romic I, Silovski H, Mance M (2021). Psychological effects of double Crisis (COVID-19 pandemic and Earthquakes) on Croatian Medical Students. Psychiatr Danub.

[48] Amici P (2021). Intolerance of uncertainty: from Transdiagnostic Model to Clinical Management. Psychiatr Danub.

[49] Jing GP, Katz CL (2021). An update on psychotic spectrum disorders and Disasters. Curr Opin Psychiatry.

[50] Ferrara M, Curtarello EMA, Gentili E (2023). Sex differences in schizophrenia-spectrum diagnoses: results from a 30-year health record registry. Arch Womens Ment Health.

[51] de Portugal E, González N, Miriam V, Haro JM, Usall J, Cervilla JA (2010). Gender differences in delusional disorder: evidence from an outpatient sample. Psychiatry Res.

[52] Grossman LS, Harrow M, Rosen C, Faull R (2006). Sex differences in outcome and recovery for schizophrenia and other psychotic and nonpsychotic disorders. Psychiatr Serv.

[53] Mihaljevic-Peles A, Sagud M, Filipcic IS, Grosic V, Pedisic I, Emsley R (2016). Remission and employment status in schizophrenia and other psychoses: one-year prospective study in Croatian patients treated with risperidone long acting injection. Psychiatr Danub.

[54] van der Werf M, Hanssen M, Köhler S, Verkaaik M, Verhey FR, van Winkel R, van Os J, Allardyce J, RISE Investigators (2014). Systematic review and collaborative recalculation of 133,693 incident cases of schizophrenia. Psychol Med.

[55] Sommer IE, Tiihonen J, van Mourik A, Tanskanen A, Taipale H (2020). The clinical course of schizophrenia in women and men-a nation-wide cohort study. NPJ Schizophr.

[56] Ujhelyi Gomez K, Corcoran R, Ring A (2022). Characteristics of mental health stability during COVID-19: an online survey with people residing in a city region of the North West of England. PLoS ONE.

[57] Caldiroli A, Capuzzi E, Tringali A, Tagliabue I, Turco M, Fortunato A (2022). The psychopathological impact of the SARS-CoV-2 epidemic on subjects suffering from different mental disorders: an observational retrospective study. Psychiatry Res.

